# Chondroitin Sulfate-Based Cryogels for Biomedical Applications

**DOI:** 10.3390/gels7030127

**Published:** 2021-08-26

**Authors:** Sahin Demirci, Mehtap Sahiner, Betul Ari, Aydin K. Sunol, Nurettin Sahiner

**Affiliations:** 1 Department of Chemistry, Faculty of Sciences & Arts, and Nanoscience and Technology Research and Application Center (NANORAC), Canakkale Onsekiz Mart University Terzioglu Campus, Canakkale 17100, Turkey; sahindemirci@gmail.com (S.D.); betullcan@gmail.com (B.A.); 2 Faculty of Canakkale School of Applied Science, Canakkale Onsekiz Mart University Terzioglu Campus, Canakkale 17100, Turkey; sahinerm78@gmail.com; 3 Department of Chemical & Biomedical Engineering, and Materials Science and Engineering, University of South Florida, Tampa, FL 33620, USA; asunol@usf.edu; 4 Department of Ophthalmology, University of South Florida, Tampa, FL 33620, USA

**Keywords:** chondroitin sulfate, croyogel, metal chelating, blood compatibility, fibrinogen effect

## Abstract

Cryogels attained from natural materials offer exceptional properties in applications such as tissue engineering. Moreover, Halloysite Nanotubes (HNT) at 1:0.5 weight ratio were embedded into CS cryogels to render additional biomedical properties. The hemolysis index of CS cryogel and CS:HNT cryogels was calculated as 0.77 ± 0.41 and 0.81 ± 0.24 and defined as non-hemolytic materials. However, the blood coagulation indices of CS cryogel and CS:HNT cryogels were determined as 76 ± 2% and 68 ± 3%, suggesting a mild blood clotting capability. The maximum% swelling capacity of CS cryogel was measured as 3587 ± 186%, 4014 ± 184%, and 3984 ± 113%, at pH 1.0, pH 7.4 and pH 9.0, respectively, which were reduced to 1961 ± 288%, 2816 ± 192, 2405 ± 73%, respectively, for CS:HNT cryogel. It was found that CS cryogels can hydrolytically be degraded 41 ± 1% (by wt) in 16-day incubation, whereas the CS:HNT cryogels degraded by 30 ± 1 wt %. There is no chelation for HNT and 67.5 ± 1% Cu(II) chelation for linear CS was measured. On the other hand, the CS cryogel and CS:HNT cryogel revealed Cu(II) chelating capabilities of 60.1 ± 12.5%, and 43.2 ± 17.5%, respectively, from 0.1 mg/mL Cu(II) ion stock solution. Additionally, at 0.5 mg/mL CS, CS:HNT, and HNT, the Fe(II) chelation capacity of 99.7 ± 0.6, 86.2 ± 4.7% and only 11.9 ± 4.5% were measured, respectively, while no Fe(II) was chelated by linear CS chelated Fe(II). As the adjustable and controllable swelling properties of cryogels are important parameters in biomedical applications, the swelling properties of CS cryogels, at different solution pHs, e.g., at the solution pHs of 1.0, 7.4 and 9.0, were measured as 3587 ± 186%, 4014 ± 184%, and 3984 ± 113%, respectively, and the maximum selling% values of CS:HNT cryogels were determined as 1961 ± 288%, 2816 ± 192, 2405 ± 73%, respectively, at the same conditions. Alpha glucosidase enzyme interactions were investigated and found that CS-based cryogels can stimulate this enzyme at any CS formulation.

## 1. Introduction

Chondroitin sulfate (CS), a glucosamine and an important component of cartilage, can be obtained from various sources, including cartilage of cow or shark. CS is a glycosaminoglycan (GAG) of a sulfated polysaccharide formed by multiple repetitions of the disaccharide units with the combination of D-Glucuronic acid and N-acetyl-D-galactosamine. Together with collagen, it forms the main component of cartilage [1]. CS is abundant in all invertebrates and is found in the cartilage, skin, blood vessels, ligaments and tendons of all mammals [2]. With its sulfate and carboxyl groups, it can bind different molecules such as growth factors, cytokines, chemokines, and adhesion molecules [2]. Additionally, CS is known for its in vivo and in vitro antioxidant properties, and Fe(II) and Cu(II) binding studies have been widely carried out to show the antioxidant activity because of the chelating capacity of CS with the transition metals ions such as Cu(II) and/or Fe(II) ions [3,4]. It was shown that CS could inhibit the α-amylase enzyme, which is an enzyme that is significant in diabetes [1]. α-glucosidase and alpha-amylase are hydrolysis enzymes that break disaccharide and polysaccharides. The inhibitors of these enzymes can play a significant role in Diabetes Mellitus type 2 [5]. In addition to its and biocompatible properties, CS is also approved as a wound dressing material and has been used in the supportive therapy for joint pains and atherosclerosis [6].

Halloysite nanotubes (HNT) are natural clays in the form of open-ended tubes, generally containing Al_2_O_3_ with Al(OH)_3_ on the inner surface and SiO_2_ with Si-OH groups on the outer surface, which are aluminosilicates [7,8]. The natural HNT clay, because of its abundance and physical and chemical properties, has a wide range of possibility in the construction industry and biomedical fields. HNT clays are used in cosmetics, filling materials, insulation materials, electrochemical sensors, and the use in the biomedical fields is increasing continuously [9,10,11]. Additionally, HNT is a unique entrapment material or carrier for loading and releasing of drugs, enzymes, DNA, antioxidant agents, proteins and so on [12,13].

Cryogels are supermacroporous structures that are synthesized under the freezing point of the solvent, e.g., for water below (sub) zero temperature [14]. They are prepared by polymerization or crosslinking or simultaneous polymerization and crosslinking of monomeric or polymeric precursors [15]. Cryogels harness superior properties, e.g., fast reaction to the change in their environment, higher mechanical strength, and better elasticity due to their interconnected super-macroporous structures in comparison to conventional hydrogels [16,17,18]. For example, cryogels’ maximum swelling time is comparably faster than that of a common hydrogel. Additionally, the higher porosity makes them very useful for tissue engineering and separation purposes [19]. Recently, the preparation of cryogels from natural, synthetic monomers or polymers or biohybrid as precursors for biomedical use is attracting significant interest [20,21,22]. Many cryogels were prepared from natural polymer-based materials and molecules because of their nontoxicity, biodegradability, biocompatibility and non-immunogenicity [23,24]. Additionally, because of their tunable pore sizes and with desirable functional groups for different molecules, they can be a reactor or cell bioreactor [25]. For example, sulfonated cryogel scaffolds can be used for focal delivery in ex vivo brain tissue cultures. In a study by Eigel et al., sulfonated cryogel scaffolds were prepared and it was demonstrated that they can be used for focal delivery in ex vivo brain tissue cultures [26]. Another study by Kuo et al. reported the preparation of gelatin/chondroitin-6-sulfate/hyaluronan-chitosan cryogels and showed that these materials are suitable scaffolds for cartilage tissue engineering [27]. Moreover, Han et. al. reported a gelatin-based cryogel containing methacrylated chondroitin sulfate for tissue engineering and revealed that the cryogel showed significant cartilage tissue compatibility [28]. CS-based hydrogels were reported for bone tissue engineering and it was shown that the negatively charged sulfate groups in CS can readily bind calcium and phosphate ions to promote biomineralization [29].

In this study, CS-based cryogels were synthesized in cryogenic conditions using divinyl sulfone crosslinker and HNT as natural clay as a filling material. The swelling behaviors and hydrolytic degradation behaviors of CS-based cryogels were studied. Furthermore, the blood compatibility, metal chelating capacity, and enzyme interaction properties of the prepared CS-based cryogels for potential use in biomedical applications were examined.

## 2. Results and Discussion

### 2.1. Synthesis and Characterization of CS-Based Cryogels

**Figure 1:**
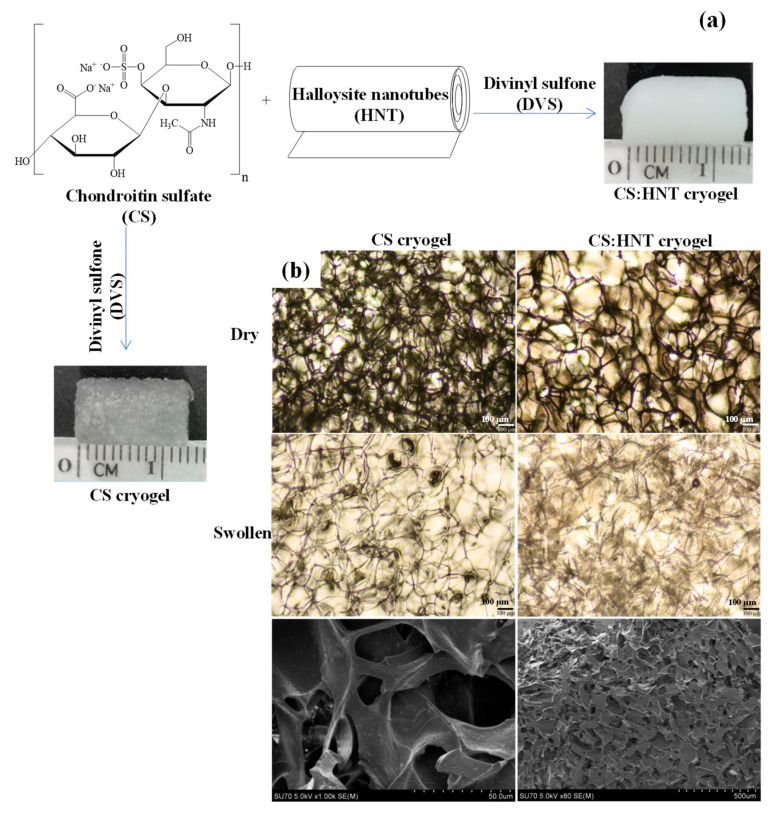
(**a**) Schematic representation of cryogels synthesis, and (**b**) the optic microscope images of dry and swollen cryogels and the corresponding SEM images of the croyogels.

CS and CS:HNT cryogels were synthesized by cryo-gellation method at −18 °C with DVS as crosslinker. The schematic representation of CS and CS:HNT cryogel synthesis and digital camera images of the corresponding cryogels are presented in Figure 1a. Hydroxyl groups of CS were crosslinked with the vinyl groups of the DVS in a basic condition to obtain super porous CS and CS:HNT cryogels. While the CS cryogel is sem-transparent, it turns white upon the incorporation of HNT into the network structure.

Figure 1b shows optical microscope images of CS cryogel and CS:HNT cryogel (first row dry form and second row swollen forms) and the SEM image of the last row CS cryogel. Optical microscope images show irregular pores of about 100 µm in size for both types of cryogel. From the examination of the SEM image of the CS cryogel, it is obvious that macroporous structures with wide pore sizes ranging from 50 to a few hundred nm are present. CS hydrogel was previously synthesized via photocrosslinked with glycidyl methacrylate [30], and nanoparticles of CS with Chitosan as ionically crosslinked particles have been previously reported [31]. However, to the best of our knowledge, no one has reported the synthesis of CS cryogels, and the CS-based cryogels were synthesized for the first time with this investigation.

**Figure 2:**
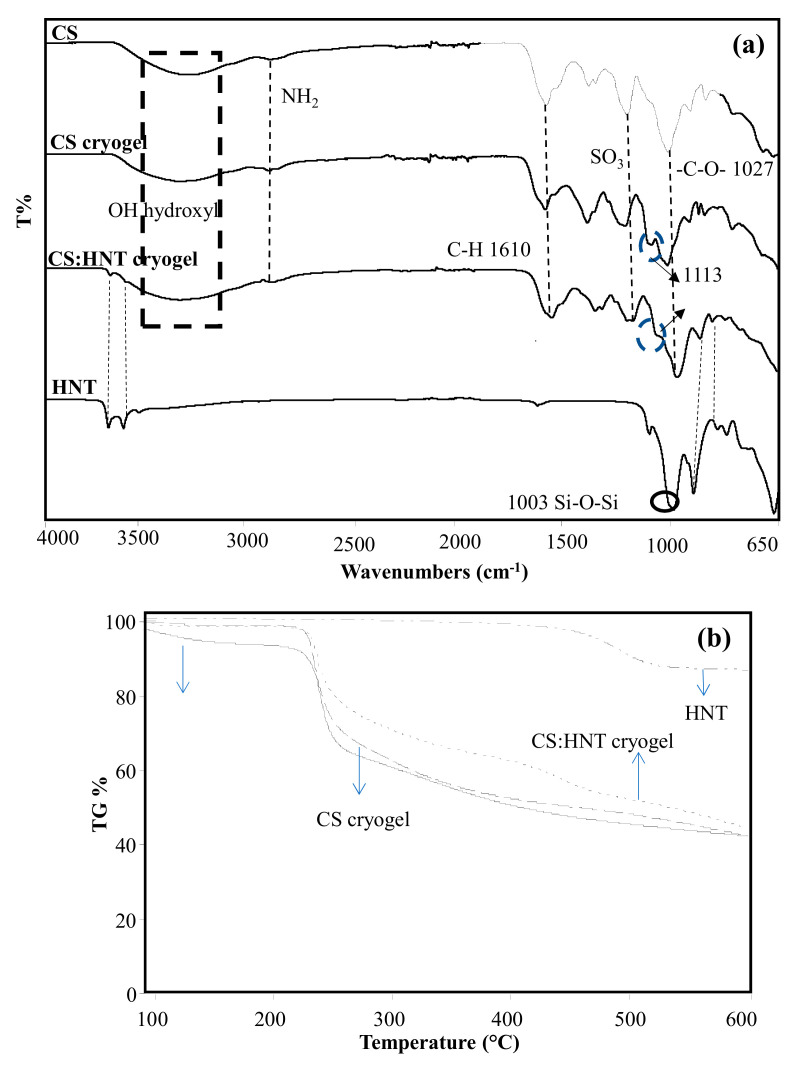
(**a**) FT–IR spectra and (**b**) thermal degradation behaviors of CS, HNT, CS cryogel and CS:HNT cryogel.

The FT-IR spectra of CS, CS cryogel, HNT, CS: HNT cryogel composites were illustrated in Figure 2a. The FT-IR spectrum of CS has a hydroxyl group at 3369 cm^−1^ and the symmetric NH-stretching at 2928 cm^−1^, aliphatic C-H at 1608 cm^−1^, C=O at 1560 cm^−1^, 1230 cm^−1^ for O-SO_3_ group and a carboxyl group at 1061 cm^−1^ peaks matched with the references [31]. The FT-IR spectrum of CS cryogel have OH stretching vibrations between 3300 and 3100 cm^−1^, asymmetric stretching of free carboxylate groups at 1605 cm^−1^, and S=O stretching from DVS at 1113 cm^−1^ wavenumbers. The FT-IR peak of HNT has peaks for Si-OH stretching at 3693 and 3620 cm^−1^, and for Al-OH bending at 907 cm^−1^, and for Si-O-Si stretching at 1003 and 791 cm^−1^ wavenumbers. Additionally, the FT-IR spectrum of CS: HNTs composite cryogel also has shown peaks for both CS and HNTs, such as peaks at 1610 cm^−1^ wavenumbers for CS cryogels, and peaks at 3693 and 3620 cm^−1^ wavenumbers from HNTs.

The thermal degradation behaviors of CS, HNT, CS cryogel, and CS:HNT cryogel were also examined, and their thermograms are given in Figure 2b. The thermal degradation of HNT shows a one-step degradation profile, starting with a 2% weight (for water moisture) loss at 450 °C and ending with a 13% weight loss at 600 °C. CS and CS cryogel also showed dissimilar thermal degradation behavior. CS and CS cryogel degraded in two stages; the initial stage of degradation with 32% weight loss at between 103–253 °C and 30% weight loss between 226–258 °C, respectively. The second stage of degradation began at 253 °C for both CS and 258 °C for CS cryogel and ended with a total mass loss of 59% at 600 °C for both. CS:HNT cryogel also initially exhibited similar thermal degradation behavior to CS cryogel. The first degradation stage begins with a 30% weight loss in the 226–307 °C range. Additionally, the second degradation stage started at 307 °C and ends with a weight loss of 54% at 600 °C.

**Figure 3:**
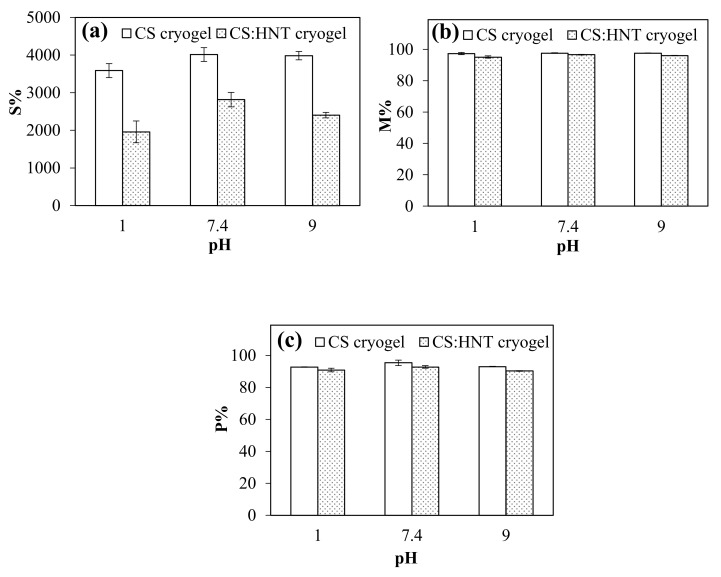
(**a**) Swelling ratio (S%), (**b**) moisture content (M%) and (**c**) porosity (P%) of CS and CS:HNT cryogel at pHs 1, 7.4 and 9.

Swelling ability, moisture content and porosity of CS cryogel and CS:HNT cryogels were determined at pH 1.0, pH 7.4 and pH 9.0 buffer solutions, and the results are presented in Figure 3a–c, respectively. As seen from Figure 3a, the higher maximum swelling behaviors were obtained for of CS cryogels, 3587 ± 186%, 4014 ± 184%, and 3984 ± 113%, at pH 1.0, pH 7.4 and pH 9.0, respectively, and the maximum selling % values of CS:HNT cryogels were determined as 1961 ± 288%, 2816 ± 192, 2405 ± 73%, respectively, under the same conditions. The acidic group ionization is higher at higher solution pHs; the higher s% values for CS and CS:HNT cryogels were expected.

Figure 3b gives the percent moisture content (M%) of the cryogels at pHs 1.0, 7.4 and 9.0. Independent of the pH conditions, the moisture content (M%) of CS cryogel and CS:HNT cryogels are in 95–97% range suggesting that all the cryogels have fairly good water retention ability.

As presented in Figure 3c, the porosity percentage (P%) of CS cryogel and CS:HNT cryogels at pH 1.0, pH 7.4 and pH 9.0 were calculated as 92 ± 1%, 95 ± 2% and 93 ± 1% cryogel, and 90 ± 1%, 93 ± 1% and 91 ± 1% for CS:HNT cryogel, respectively. The P% results corroborate the maximum swelling studies, e.g., at lower swelling rates, the porosity and pore volume were reduced. Therefore, the smaller porosity of the CS:HNT cryogel is expected in comparison to bare CS cryogels.

The pore volume (Vp), gel density and gel content% of CS cryogel and CS:HNT cryogels were calculated and the corresponding results are summarized in Table 1. The pore volume of CS cryogel and CS:HNT cryogels were calculated and found as 31.55 ± 6.37 mL·g^−1^ and 17.50 ± 3.15 mL.g^−1^, respectively. Additionally, the gel densities of CS cryogel and CS:HNT cryogel were calculated as 0.11 ± 0.03 g. mL^−1^, and 0.17 ± 0.09 g·mL^−1^, respectively. The gel contents of the same cryogels were also calculated and found as 60 ± 3.31% and 76 ± 0.84%, respectively. The findings revealed that HNT caused a decrease in pore volume, an increase in gel density, and an increase in gel content by tightening/filling the pores of the 3D network structures with another porous material (HNT itself).

### 2.2. Hydrolytic Degradationof CS-Based Cryogels

**Figure 4:**
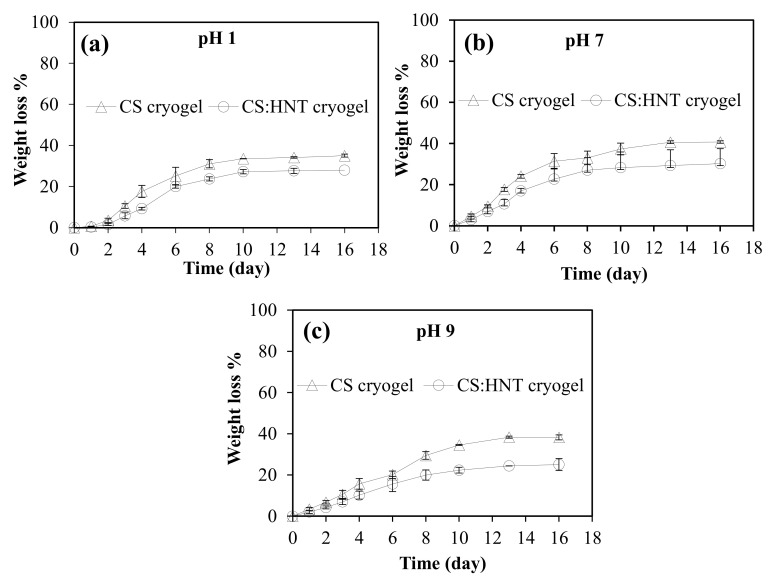
Hydrolytic degradation of CS cryogel and CS:HNT cryogels at pH (**a**) 1, (**b**) 7.4 and (**c**) 9.

Hydrolytic stability is a crucial parameter for materials to be used in biomedical applications. To determine hydrolytic stability of CS and CS:HNT cryogels, the degradation behaviors at three different pH conditions such as pH 1.0 (about gastric conditions), pH 7.4 (physiological conditions) and pH 9.0 (about intestine conditions) were investigated at 37.5 °C. The weight change in CS and CS:HNT is presented in Figure 4a–c at pHs 1, 7.4 and 9, respectively. Irrespective of the pH condition, CS and CS:HNT cryogels were found to degrade almost linearly up to 6 days, and the degradation process continued slowly up to 16 days until a plateau region was reached. As seen in Figure 4a, the first rapid degradation process of CS and CS:HNT cryogels at pH 1.0 results in a weight loss of 25 ± 4% and 19 ± 1%, respectively, after 6 days. Additionally, then the degradation process continued slowly, and after 16 days, it increased to about 35 ± 1% and 28 ± 1% for CS and CS:HNT cryogels, respectively.

Figure 4b shows the degradation behaviors of the cryogels at pH 7.4. It is also clear that CS and CS:HNT cryogels degraded with the total of weight losses of 31 ± 4% and 23 ± 5%, respectively, after the initial linear rapid degradation process up to 6 days, and then the weight losses were reached to 41 ± 1 wt % and 30 ± 1 wt %, respectively, after 16 days, with reaching to almost plateau. Finally, Figure 4c reports the degradation behavior of cryogels at pH 9.0. As can be seen, the degradation process of CS and CS:HNT cryogels at pH 9.0 revealed an almost linear degradation up to 10 days and results in a weight loss of 20 ± 2% and 16 ± 4% after 6 days. After 10 days, the degradation process slowed and after 16 days, it increased to about 38 ± 1% and 25 ± 3%. Therefore, it can be clearly stated that at all pH values the degradation is linear up to 6 days and then slightly increases with time. Additionally, CS and CS:HNT cryogels have shown maximum degradation behavior at pH 7.4 with about 40 and 30 wt % losses.

### 2.3. Hemoompatibility Assay of Cryogels

In biological application, one of the parameters to be considered for the materials is hemocompatibility. Hence, a hemocompatibility assay of the cryogels was carried out by hemolysis, blood coagulation, as well fibrinogen tests for the potential use in blood contacting applications.

**Figure 5:**
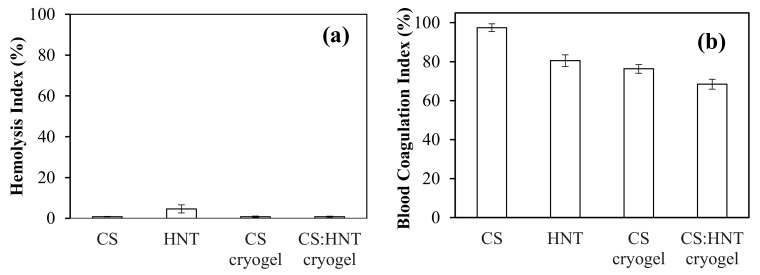
Hemocompatibility test of CS, HNT, CS cryogel and CS:HNT cryogels, (**a**) hemolysis test and (**b**) blood coagulation test.

Hemolysis is red blood cell destruction, and hemocompatibility of a material is classified as follows: 0–2% hemolysis ratio is classed as non-hemolytic, 2–5% hemolysis as less hemolytic, and >5% hemolysis as hemolytic, based on their hemolysis index [5]. The hemolysis index results of CS, HNT, CS cryogel, CS:HNT cryogels are presented in Figure 5a. The results show that CS, CS cryogel and CS:HNT cryogel are non-hemolytic, whereas HNT is found to be hemolytic, implying that HNT can damage red blood cells. However, by the inclusion of HNT into CS cryogel (CS:HNT) the prepared composite cryogels become non hemolytic, e.g., do not damage red blood cells.

Blood coagulation index assay (another hemocompatibility test) was also carried out for CS, HNT, CS cryogel, and CS:HNT cryogel weighing 10 mg, and the results are illustrated in Figure 5b. The blood coagulation indices of the CS molecule, HNT, CS cryogel, and CS:HNT cryogel were found as 97 ± 2, 80 ± 3, 76 ± 2, and 68 ± 3%, respectively. As can be seen, the CS molecule and CS cryogel did not affect blood coagulation, whereas HNT and CS:HNT cryogel caused some blood coagulation. In conclusion, CS and CS:HNT cryogels are materials that do not harm red blood cells and have a slight interfering effect on blood clotting. Therefore, in blood contact application of CS and/or CS:HNT, the amount of CS-based cryogel materials should be ≤10 mg.

### 2.4. The Interaction of CS-Based Cryogel with Fibrinogen

**Figure 6:**
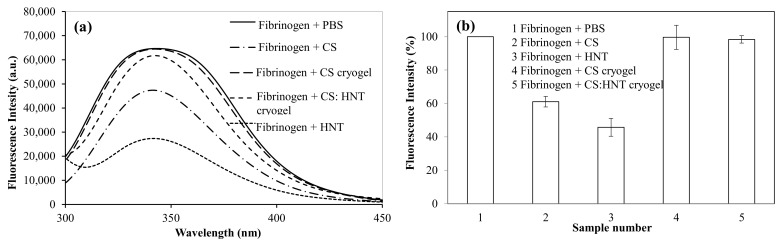
(**a**) The reduction in the fluorescent intensity of fibrinogen upon interaction with CS, HNT, CS cryogel and CS:HNT cryogel at the concentration of 1.0 mg/mL, and (**b**) the percentage change in emission intensity of CS, HNT, CS cryogel and CS:HNT cryogel interacting with fibrinogen at 341 nm wavelength (excitation wavelength: 280 nm).

Fibrinogen is a blood protein [32], and it plays a significant role in the blood clotting mechanisms. One of the mechanisms is the conversion of soluble fibrinogen to insoluble fibrin that forms a hemostatic clot [33]. There is also a linear relationship between the change in the structure of fibrinogen and coagulation [32], and fibrinogen has a fluorescence property due to the tryptophan residues in its structure. CS and CS-based cryogel samples at 1.0 mg/mL concentration in PBS interacted with a fibrinogen solution (0.2 mg/mL), and the change in fluorescence intensity was observed. In Figure 6, the fluorescence intensity of fibrinogen upon interacting with various CS-based samples is shown. As seen in Figure 6a, the fibrinogen has a fluorescent emission peak at 341 nm with an excitation wavelength of 280 nm. When fibrinogen interacted with CS samples, the peak intensity at 341 nm was reduced and the calculated reduction as a percentage in the fluorescent intensity is given in Figure 6b. The fluorescence intensity of fibrinogen upon interacting with CS samples was listed as CS cryogel > CS: HNT cryogel > CS > HNT. In the fibrinogen interaction of CS cryogel and CS: HNT cryogel, the percentage of fluorescence intensity was determined as 99.6% and 98.3%, while 61.1% and 45.7% fluorescent intensities with CS and HNT, respectively, were observed. So, CS cryogel has almost no effect on the fluorescence of fibrinogen, while CS and HNT greatly reduce the fluorescence of fibrinogen. Therefore, the CS-based cryogels seem more suitable for conditions where arterial and venous materials or clots are not desired.

### 2.5. Metal Chelating Activities of CS-Based Cryogels

**Figure 7:**
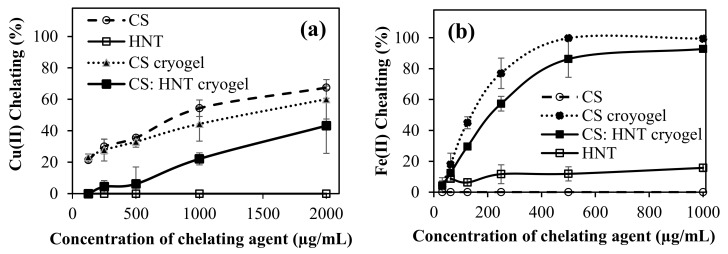
(**a**) Cu(II), and (**b**) Fe(II) chelating capability of CS, HNT, CS cryogel and CS: HNT cryogels.

Cu(II) and Fe(II) ions’ chelating capability of CS-based cryogels were studied. The chelating capacity of Cu(II) ion and Fe(II) ion was given as the percentages of metal-chelating in Figure 7a,b, respectively. As can be seen, the Cu(II) chelating capacity was the highest, with linear CS, and it increased with concentration. It was also determined that at 2000 µg/mL concentration, CS chelated 67.5 ± 1% of Cu(II) ions, whereas the CS cryogel and CS:HNT cryogel at the same concentration showed the Cu(II) chelation capability of 60.1 ± 12.5, and 43.2 ± 17.5%, respectively. Interestingly, HNT did not show any Cu(II) chelation capability at any of the concentrations, justifying the reduction in CS:HNT cryogel Cu(II) ion binding. It was reported that D-penicillamine: (S)-2-amino-3-mercapto-3-methylbutanoic acid, Tetrathiomolybdate, triethylenetetramine dihydrochloride, 5,7-Dichloro-2[(dimethylamino)methyl]quinolin-8-ol, and 2,3-Dimercaptosuccinic acid are some of the Cu (II) chelators [34]. All these chemicals contain functional groups such as -OH, -SH, and N-containing structures. These functional groups can readily chelate with Cu(II) ions. In CS, there are plenty of -OH and -SO_3_H exist, which can participate in chelating with Cu(II) ions. The lesser extent of chelation capability of CS cryogel than CS polymer can be explained with the existence of some DVS groups coming from the crosslinker that may reduce the chelation capability of CS cryogels.

In the Fe(II) chelating capacity test, the concentrations of the CS-based samples were varied between 2000 and 31.25 µg/mL. Interestingly, CS did not show any Fe(II) chelation capability at any concentration. However, CS cryogel and CS: HNT cryogel demonstrated a concentration-dependent increase in Fe(II) chelation capability. At 500 µg/mL concentration, the CS cryogel chelated 99.7 ± 0.6% of the Fe(II), while the CS: HNT cryogel chelated 86.2 ± 4.7% of Fe(II), and HNT, however, chelated only 11.9 ± 4.5% of Fe(II) at the same concentration. As Fe(II) chelating activity was examined, it was observed that CS did not chelate with Fe(II) ions. On the other hand, CS cryogel revealed some chelating capability for Fe(II) ions. This may be due to the three-dimensional structure of the cryogels in comparison to the linear CS polymer, as well as to the existence of DVS groups that may have some kind of affinity to bind with Fe(II) ions. The similar behavior was also observed for tannic acid and poly(tannic acid) particles; the particle form of tannic acid afforded significant increase in Fe(II) cheating capability [35]. Moreover, it was also reported that a flavonoid, rutin molecule showed no binding ability for the Fe(II) ion, whereas the micro/nanogels form of rutin had considerable Fe(II) binding ability [5]. Linear CS, which chelated Cu(II) the highest, did not show any chelating capability with the Fe(II) ion. It has been observed that CS has a selective property against Cu(II) ions. Additionally, the non-Fe(II) binding capability of linear CS is significantly improved by turning the linear CS into cryogel forms and cryogel composite forms with HNT, because the 3-D structure and existence of DVS groups in cryogel enable the Fe(II) complexion. The imbalance of metal ions leads to the accumulation of amyloid-β peptide (Aβ) proteins, causing neurological diseases such as Alzheimer’s and Parkinson’s [36]. There are many studies in the literature to prevent Aβ aggregation by chelating Cu(II) and Fe(II) ions [37]. In the process of leading to iron overload-related diseases, metallic iron creates highly toxic Reactive Oxygen Species (ROS) [38]. Here, the obtained results indicate that CS cryogels can be potentially used as a metal chelator in the treatment of some neurological diseases.

### 2.6. α-Glucosidase Interaction with CS-Based Cryogel

**Figure 8:**
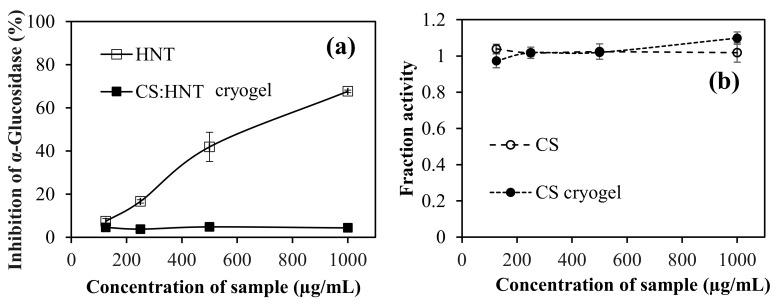
(**a**) Inhibition of α-glucosidase percentage of HNT and CS:HNT cryogel, and (**b**) α-glucosidase fraction activity of CS and CS cryogels.

The α-glucosidase (E.C.3.2.1.20) enzyme is one of the hydrolysis enzymes in the small intestine that break down disaccharides linked by (1–4) alpha bonds [39]. Additionally, α-amylase is another enzyme that breaks down polysaccharides linked by alpha bonds [40]. These two enzymes also take part in the breakdown of sugars in humans. Therefore, they play an important role in the absorption of sugars in the digestive tract, as erythrocytes can only absorb monosaccharides. The inhibitors of these enzymes are used in the treatment of type 2 diabetes patients [41]. In the literature, it has been reported that CS inhibits the alpha amylase [1]. Therefore, the inhibition of α-glucosidase by HNT clay and CS:HNT cryogels, and linear CS and CS cryogel is tested at pH 6.9 phosphate buffer, and the results are shown in Figure 8a,b, respectively.

As shown in Figure 8a, CS:HNT inhibited only 4.4 ± 5.0% of enzymes at 1000 µg/mL concentration, while HNT inhibited 67.7 ± 0.6%. On the other hand, CS and CS cryogel, did not inhibit α-glucosidase at any concentration up to 1000 µg/mL, as can be seen in Figure 8b, in which the fraction activity is about 1. So, it is obvious that the enzyme activity was stimulated by CS in any formulation. Some compounds that consist of phenolics can inhibit the α-glucosidase enzyme, while some do not [39]. For example, rosmarinic acid was shown to inhibit the α-glucosidase enzyme whereas, some phenolic compounds, such as naringin and rutin, did not inhibit α-glucosidase [5,42,43]. Additionally, acarbose has a sugar unit (e.g., linked glucose) used as α-glucosidase inhibitor standard, whereas CS also has a sugar unit, and it can not inhibit α-glucosidase. The inhibitors block α-glucosidase enzyme activity by completely blocking access to its active site. In this study, it was assessed that CS did not block the active ends of α-glucosidase. In some glucose storage diseases, the body becomes incapable of converting glycogen into glucose [44,45]. An acid alpha-glycoside deficiency causes pompe disease, which is triggered by glycogen build-up in the cells. Biochemical reactions in the body are activated by these enzymes. In a healthy person with normal enzyme activity, the function of this enzyme is to destroy the complex sugar melodies stored in lysosomes in cells. Therefore, insufficient or defective enzymes cannot eliminate the complex sugar molecules, so storage occurs [46]. Therefore, the presence of CS and CS cryogel does not affect the fractional activity of α-glucosidase and can be used in such applications.

On the other hand, in cases where co-inhibition of α-glucosidase and α-amylase enzymes is desired, this effect is created by adding various drugs or natural inhibitors. In patients with type 2 diabetes, materials that provide inhibition of both enzymes (α -glucosidase and α-amylase) may be needed.

## 3. Conclusions

As the synthesized CS-based cryogels are biodegradable and hemocompatible, it is suggested here that CS-based cryogels can be safely used for tissue engineering, or bioreactor, cell separation or scaffolding materials. As cryogels possess interconnected super porous material, they can also be used for neural precursor cell microcarriers [47], and are useful for nerve guidance conduits, which are designed to guide nerves. The CS-based cryogels provide great potential in not only neurological applications due to porosity, permeability, adhesion of growth factors, multi-channel structure and polymer binding ability [48], but also metal ions such as Cu(II) and Fe(II) binding capability. The super porous CS-based cryogels were shown to be readily prepared with another natural porous clay material such as HNT, and these composite cryogels can be very effective as different cell growth and differentiation media and in the transport of many molecules as microcarriers. Additionally, the prepared CS-based cryogel can be very useful in the prevention and treatments of some neurological diseases due to its chelating capability of different metal ions.

## 4. Materials and Methods

### 4.1. Materials

Chondroitin sulfate A sodium salt (CS, 475,379 g/mol per repeating unit, Alfa Aesar, 90%) divinyl sulfone (DVS, Sigma Aldrich, St. Louis, MO, USA), Cupric sulphate pentahydrate (CuSO_4_·5H_2_O), and pyrocatechol violet (PV, 3,3′,4-trihydroxyfuchsone-2″-sulfonic acid) are used in Cu(II) chelating studies. 3-(2-pyridyl)-5,6-diphenyl-1,2,4,-triazine-4′,4″-disulfonic acid sodium salt (ferrozine, ≥98%, Santa Cruz Biotechnology, Dallas, TX, USA) and iron(II) sulfate heptahydrate (FeO_4_S∙7H_2_O > 99.5%, ACS reagent, Acros Organics, Geel, Belgium) were used in Fe(II) chelating activity studies. Bovine fibrinogen (Alfa Aesar, Haverhill, MA, USA) was used for fibrinogen interactions of CS-based materials. α-D-glucosidase from Saccharomyces cerevisiae (Sigma, 100 unit/g), p-nitrophenyl-α D-glucopyranoside (RPI), and reduced glutathione (Across) were used for enzyme studies.

### 4.2. Synthesis and Characterisation of Cryogels

CS weighing 0.08 g was dissolved in 3.2 mL of 0.2 M NaOH solution and divinyl sulfone (DVS) 100% (based mole ratio of CS) was added on the solution as a crosslinker, then the mixture was vortexed and filled into 5 mm plastic pipettes and kept in a deep freezer at −18 degrees for 2 days. Then, the pipettes were taken out of the freezer and brought to room temperature and the resulting cryogels were cut into 5 mm in lengths, and washed 3 times with excess amount of DI water in a 100 mL beaker to remove the unbounded CS. Finally, the cryogels were freeze-dried, before further use.

CS:HNT cryogels were synthesized in a similar way as mentioned above, but 0.04 g HNT was added into the cryogel precursor solutions.

The structural characterization of the CS, HNT, CS cryogel, and CS:HNT cryogel were carried out using FT-IR spectroscopy (FT-IR, Nicolet iS10, Thermo, Waltham, MA, USA) in the spectral range of 4000–650 cm^−1^ at a resolution of 4 cm^−1^ using the ATR technique.

The thermogravimetric analyzer device (TGA, SII TG/DTA 6300, Tokyo, Japan) was used to assess the thermal stability of the prepared cryogels. About 2 mg of lyophilized sample of cryogels was placed in a ceramic pan. Thermal degradation of the samples was carried out in N_2_(g) atmosphere at 100 mL/min flow rate, 5 °C/min heating rate, in 80–600 °C temperature range. The thermal degradation graph of the samples was created by plotting a temperature graph against the weight loss.

Morphological structures of synthesized CS cryogel and CS:HNT cryogel were investigated using optic microscope (Olympus, BX53F, Tokyo, Japan) and Scanning Electron Microscopy (SEM, Hitachi SU-70, Tokyo, Japan). A thinly cut lyophilized piece of cryogel was placed on the microscope slide and optical microscope images were taken under 4× magnification. In order to obtain the optical images of the cryogels in swollen state, a few drops of distilled water were dropped on the cryogel piece, and the images of the swollen cryogel piece were taken at the same magnification. In order to acquire the SEM images of cryogels, first the lyophilized cryogels were placed on SEM stubs and then coated with Au-Pd under vacuum, and then the SEM images were taken under accelerating voltages of 5.0 kV.

### 4.3. Swelling Behavior and Hydrolytic Degradation of Cryogels

Swelling behavior of CS and CS:HNT cryogels at different pH conditions were investigated gravimetrically. Briefly, about 15 mg of lyophilized CS and CS:HNT cryogel pieces were weighed. Then, the cryogel pieces were swollen for 2 min in 10 mL volumes of pH 1.0, 7.4, and 9.0 buffer solutions (PBS). The swollen cryogels were taken out and blot dried gently to remove excess surface water via filter paper, and then re-weighed. The swelling ratio% and moisture capacities% of cryogels were calculated using Equations (1) and (2), respectively. To determine the porosity of the cryogels, the same swollen cryogels were squeezed and re-weighed. The percentage of porosity (*P%*) of cryogel samples was calculated using Equation (3).

(1) $$S\%=(m_{s}-m_{l})/(m_{l}\times 100)$$

(2) $$M\%=(m_{s}-m_{l})/(m_{s}\times \;100)\;$$

(3) $$P\%=(m_{s}-m_{sq})/(m_{s}\times \;100)$$

where “$m_{s}$”, “$m_{l}$” and, “$m_{sq}$” stand for swollen cryogel, lyophilized cryogel and, squeezed weight of cryogel, respectively.

To determine the pore volume (*V_p_*) of the cryogels, the lyophilized cryogel samples of known weight were kept in cyclohexane for 30 min, which is a poor solvent for these cryogels, and re-weighed. The pore volume of the cryogels was calculated using the following Equation (4).

(4) $$V_{p}=(m_{ch}-m_{l})/(m_{l}\times \;d_{c})\;$$

where, “*m_ch_*” is the weight of cryogel swollen in cyclohexane and “*d*” is the density of cyclohexane.

Density analysis and gel content terms are widely used in cryogel characterization. Therefore, the density and gel content of the cryogels were also calculated. Density of cryogels was calculated using Equation (5) upon measuring the radius and height after weighing a lyophilized cryogel.

(5) $$d=m_{l}/v_{l}$$

where, “$m_{l}$” describes lyophilized cryogel weight and “$v_{l}$” represents cylinder-shaped lyophilized cryogel volume.

The gel content was also determined by weighing the lyophilized cryogels just after preparation, then washing the cryogels exhaustively in order to remove loose polymer chains and unreacted DVS molecules. After that, the washed cryogels re-lyophilized and were weighed. The ratio between the weigh after and before washing process yields the gel content. The gel content was calculated using Equation (6).

(6) $$Gel\;content\;\%=(m_{a}/m_{b})\times \;100\;$$

where “$\;m_{a}$” is the weight of the lyophilized cryogel sample after washing and “$m_{b}$” is the weight of the lyophilized cryogel sample before washing.

The hydrolytic degradation behavior of CS and CS:HNT cryogels at different pH conditions was determined gravimetrically by calculating the weight loss observed in cryogels in the degrading medium. Cryogel samples weighing approximately 20 mg were swollen in pH 1.0, 7.4, and 9.0 buffer solutions until their maximum swelling capacity was reached. Then, these cryogels were immersed in buffer solutions corresponding to 10 mL at 37.5 °C. At certain time intervals, in the course of degradation, the cryogels were taken out of the buffer solutions and surface water was gently removed via filter paper and weighed. The weight loss% of cryogels was calculated using Equation (7).

(7) $$Weight\;loss\;\%=(100-(m_{t}/m_{i}))\times \;100$$

### 4.4. Hemocompatibilitiy Assay of Cryogels

Hemocompatibilities of cryogels were tested in vitro according to the procedure approved by Çanakkale Onsekiz Mart University Clinical Research Ethics Committee (2011-CREC-27/2020-E.2000045671). Hemocompatibility studies were conducted following the methods reported in the literature [8].

Hemolysis of CS cryogels was also tested to determine the effect of cryogel samples on red blood cells for potential use in biomedical field. The blood given by healthy and volunteer individuals was taken into blood tubes containing anticoagulants. Then, these blood tubes were mixed by slowly turning upside-down several times. Next, 2 mL of this blood sample was taken and diluted with 2.5 mL 0.9% saline solution. The diluted blood sample was taken and 0.2 mL of it was transferred to tubes containing 10 mg sample and 9.8 mL saline solution. The tubes were mixed by gently inverting and incubating at 37.5 °C for one hour. Saline solution was used as negative control and pure water as positive control. The controls were exposed to the same procedure and conditions applied to the samples. After incubation, blood solutions were centrifuged at 1320 rpm for five minute and the absorbance values of the supernatant solution were determined using UV-Vis Spectroscopy at 542 nm, where hemoglobin showed maximum absorbance. The hemolysis index% of the samples was calculated using the formula in Equation (8).

(8) $$Hemolysis\;Index\;\%=(A_{sample}-A_{saline})/\left(A_{water}-A_{saline}\right)\times 100\;$$

where, “*A_sample_*” is the absorbance value of the blood suspension containing the sample. “*A_saline_*” and “*A_water_*” are the absorbance values of the negative and positive control, respectively.

For the blood coagulation test of cryogel, samples weighing 10 mg were swollen with a few drops of 0.9% saline solution. At the same time, 0.810 mL blood sample was placed in a clean test tube, and 0.0648 mL 0.2 M CaCl_2_ aqueous solution was added to it. Then, 0.27 mL of this blood solution was taken and added to the sample swelled with saline solution. Afterward, samples contacted with blood were kept at 37.5 °C in the water bath for 10 min for incubation. After a 10-min incubation period, 10 mL of pure water was added to the samples contacted with blood, and after a short wait, the samples were centrifuged at 540 rpm for 1 min.

The non-coagulating part was taken into another tube and the total volume was completed to 50 mL with distilled water. As a negative control, 0.25 mL of blood was added directly into 50 mL of distilled water kept at 37.5 °C. Lastly, all samples were kept at 37.5 °C for one hour to complete their incubation period. The absorbance values of the pieces whose incubation period ended were recorded by using UV-Vis Spectroscopy at a wavelength of 542 nm, and the blood coagulation index% of samples were calculated following Equation (9).

(9) $$Blood\;coagulation\;Index\%=(A_{sample}/A_{control})\times \;100$$

where, “*A_sample_*” is the absorbance value of the blood suspension in contact with the sample, and “*A_control_*” is the absorbance value of the negative control.

All blood compatibility tests were performed in triplicate and the results were illustrated as hemolysis index% and blood coagulation index% graphs by calculating standard deviations.

### 4.5. Fibrinogen Interactions of Cryogels

Fibrinogen is a blood protein that interferes with thrombin to form fibrin during coagulation. The interactions of CS, HNT, CS cryogel, and CS: HNT cryogel with fibrinogen were studied. The effects of CS, HNT, CS cryogel, and CS: HNT cryogel on the fluorescence properties of fibrinogen were examined by fluorescence spectroscopy (Thermo Scientific Lumina Spectrophotometer, Waltham, MA, USA) according to the literature [32]. In PBS, a fibrinogen solution was prepared at 0.2 mg/mL concentration. A CS-based sample at 2.0 mg/mL in PBS was prepared. Sample solutions of 1 mL were mixed with fibrinogen solution in a 1:1 ratio by volume. The excitation wavelength of 280 nm was used in the fluorescence spectroscopy. The scanning range was set between 300–450 nm. The interactions of CS, HNT, CS cryogel, and CS: HNT cryogel with fibrinogen were determined in terms of the reduction in the fluorescence intensity.

### 4.6. Metal Ion Chelating Capabilitiesof CS Cryogels

Cu(II) chelating activity was carried out according to the literature, using a microplate reader (Thermo Multiscan Go, Waltham, MA, USA) at 616 nm [49]. Different concentrations of CS, CS cryogel and CS: HNT cryogel in acetate buffer pH 6 (125–2000 µg/mL) of 200 µL were put into 96-well plate. Then, 0.1 mg/mL Cu(II) solution (in acetate buffer at pH 6) of 30 µL was added to the sample solution and then there was a wait of 10 minutes. Finally, 8.5 µL of 2 mM PV solution in acetate buffer was added to the each well. As a control, 200 µL acetate buffer pH 6 solution was used.

Fe(II) cheating activity was carried out according to the literature [50]. A sample solution in DI water in 31.25–1000 µg/mL concentration with a 140 µL volume was put into a 96-well plate. Then, 20 µL of 1 mM Fe(II) was put on the sample solution, and the absorbance value was read at 562 nm with a microplate reader. Finally, 40 µL of 2.5 mM ferrozine solution was placed in the mixture and the absorbance value was read again. Cu(II) and Fe(II) chelating activity was calculated according to Equation (10).

(10) $$metal\;chelating\;activity\;\%=\left(1-\frac{\Delta Abs^{sample}}{\Delta Abs^{control}}\right)\;*\;100\;$$

### 4.7. The Effect of Cryogel on α-Glucosidase Activity

CS and CS HNT cryogel were ground in a mortar with pestle. The effect of CS, HNT, CS cryogel, and CS: HNT cryogel on α-glucosidase activity was investigated according to the literature [39,42]. In short, 70 microliters of sample suspension solution with varying concentrations (between 125 and 1000 µg/mL) was added onto 70 microliter enzyme solution and finally 70 microliter substrate solution was added. Additionally, the absorbance values at 405 nm was recorded. Additionally, the reported procedure was followed [39,42].

## Figures and Tables

**Table 1:** Pore volume (V_p_), density and gel content of CS cryogel and CS:HNT cryogel.

Cryogel Type	Vp (mL.g^-1^)	Density (g·mL^−1^)	Gel Content%	Cryogel Type
CS cryogel	31.55 ± 6.37	0.11 ± 0.03	60 ± 3.31	CS cryogel
CS:HNT cryogel	17.50 ± 3.15	0.17 ± 0.09	76 ± 0.84	CS:HNT cryogel
